# The Fallout of Catastrophic Technogenic Emissions of Toxic Gases Can Negatively Affect Covid-19 Clinical Course

**DOI:** 10.32607/actanaturae.11754

**Published:** 2022

**Authors:** G. Succi, W. Pedrycz, A. P. Bogachuk, A. G. Tormasov, A. A. Belogurov, A. Spallone

**Affiliations:** Innopolis University, Innopolis, 420500 Russia; University of Alberta, Edmonton (AB), T6G 2R3 Canada; Shemyakin-Ovchinnikov Institute of Bioorganic Chemistry RAS, Moscow, 117997 Russia; Evdokimov Moscow State University of Medicine and Dentistry, Department of Biological Chemistry, Moscow, 127473 Russia; Neurological Centre of Latium, Institute of Neurological Sciences, Rome, 00178 Italy

**Keywords:** SARS-CoV-2, COVID-19, Italy, Seveso, dioxin

## Abstract

The coronavirus D-19 (Covid-19) pandemic has shaken almost every country in the world: as we stand, 6,3 million deaths from the infection have already been recorded, 167,000 and 380,000 of which are in Italy and the Russian Federation, respectively. In the first wave of the pandemic, Italy suffered an abnormally high death toll. A detailed analysis of available epidemiological data suggests that that rate was shockingly high in the Northern regions and in Lombardy, in particular, whilst in the southern region the situation was less dire. This inexplicably high mortality rate in conditions of a very well-developed health care system such as the one in Lombardy – recognized as one of the best in Italy – certainly cries for a convincing explanation. In 1976, the small city of Seveso, Lombardy, experienced a release of dioxin into the atmosphere after a massive technogenic accident. The immediate effects of the industrial disaster did not become apparent until a surge in the number of tumors in the affected population in the subsequent years. In this paper, we endeavor to prove our hypothesis that the release of dioxin was a negative cofactor that contributed to a worsening of the clinical course of COVID-19 in Lombardy.

## INTRODUCTION

The coronavirus D-19 (Covid-19) pandemic has hit almost every country in the world as it has spread west from China to Europe, the U.S., and later South America, Africa, and the Russian Federation. At this juncture, 6.3 million COVID-19 deaths have been reported worldwide, with 380,000 of those in the Russian Federation alone. The impact of the pandemic has been particularly severe in several European countries, such as Spain, France, Belgium, the UK, and Italy, whilst other countries such as Portugal, Germany, the Scandinavian states, and Eastern Europe, in general, have had it relatively easy. Italy, in particular, has had a shockingly high mortality rate, one that significantly exceeded the death rate observed in the rest of the world.

But a closer look at the epidemiological data would suggest that this high rate was mainly concentrated just in the Northern regions, in Lombardy in particular, whilst in the southern region the clinical course of most patients was more favorable, as our group had predicted well in advance [1]. This situation is particularly troubling if the general mortality rate is compared with the one that prevailed in the previous five years. This inexplicably high mortality rate in the context of a very well-developed health care system such as the one in Lombardy – largely recognized as one of the best, if not the best, in the country [2] – certainly calls for a satisfactory explanation. Some experts have directed their attention at the potential negative role of PM-10, which are overrepresented in Lombardy [3] and some neighboring regions also significantly affected by the Covid-19 pandemic. However, if we assume that this hypothesis is sound, it becomes hard to explain why California, which is highly polluted and seriously affected by PM-10, appeared definitely less affected than other states, with New York first in mind, where the air concentration of PM-10 is lower.

In 1976 the small city of Seveso, which is relatively close to Brescia, Bergamo and Milan, became sadly known in the world for an accidental escape of dioxin. The immediate effects were mild, but that was before an increased number of tumors began to appear in the affected population in subsequent years [4]. In our work, we hypothesize that the gas escape had a negative cofactor role in the worse clinical course of the Covid-19 pandemic for patients in Lombardy.

## EXPERIMENTAL

We conducted a study correlating the distance from the epicenter of the escape of dioxin, Seveso, to the rate of mortality of the potentially affected provinces. We studied the local mortality rate from the Covid-19 infection as a percentage of the dead vs. infected patients and compared data for Lombardy with those for other world regions where a dramatic leak of toxic gases had occurred: the city of Bhopal (India), where a significant accidental toxic gas release occurred in 1980 from a local Union Carbide factory, something that was considered at the time as the worst industrial disaster in history [5, 6].

**Fig. 1:**
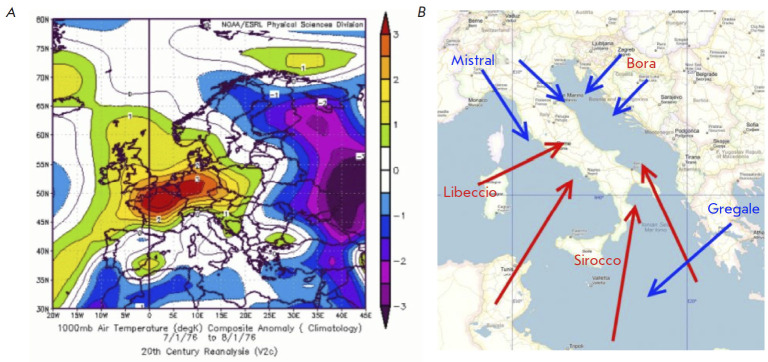
(*A*) Typical weather conditions in Northern Italy, around the Alps https://progettoscienze.com/2016/09/29/i-grandi-classici-della-scienza-libellus-de-ratiociniis-in-ludo-alee/#jp-carousel-6901 (*B*) Typical structure of winds in Italy http://sailroad.ru/article/lociya-srednej-dalmacii-chast-2

We retrieved data about the local weather conditions and winds directions at the time of the accidents and also calculated the distance between the sites of the escapes and those most clinically affected. We also analyzed the air pollution of the three sites as measured by the PM-10 concentration. The gases were in fact different: 2,3,7,8-tetrachlorodibenzodioxin in the case of Seveso [7] and methyl isocyanate in the case of Bhopal [8, 9, 10]. Nevertheless, both gases are known to be mutagenic and cancerogenic [7, 11 , 12, 13, 14, 15].

**Fig. 2:**
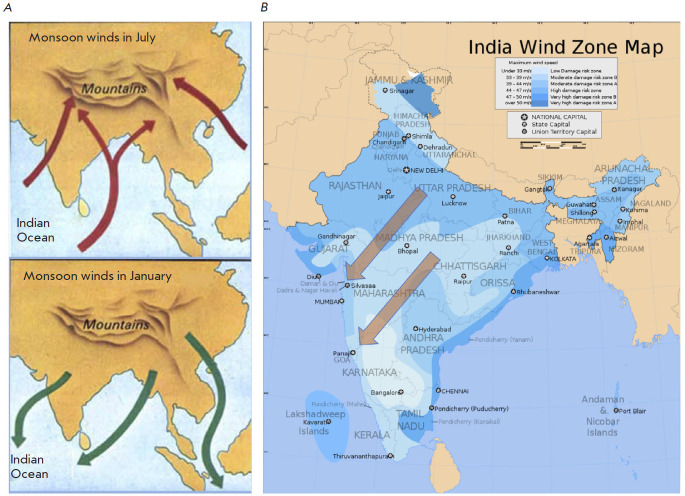
(*A*) Typical structure of Winds in India https://cloud.prezentacii.org/19/04/142027/images/screen7.jpg (*B*) Winds around Bhopal https://commons.wikimedia.org/wiki/File:India_wind_zone_map_en.svg

In both scenarios the analysis of local weather conditions allowed us to somewhat reconstruct the possible spread of the escaped gases on account of the effects of the winds. In the Seveso case, nice weather conditions in Lombardy, together with high pressure in the Alps (Fig. 1A), favored the Mistral pushing the gases south east; i.e., in the direction of Bergamo, Brescia and further south up to the western provinces of Veneto and Emilia Romagna. Some other components of the Mistral could also have pushed the gas towards eastern Piemonte and the northern part of Liguria (Fig. 1B). In the Bhopal region, where the accident occurred in December, the Monsoons typically flow from north to the southwest (Fig. 2A): so, the gases escape would have spread from the Bhopal region of Madhya Pradesh to the neighboring state of Maharashtra (Fig. 2B). As far as Italy was concerned, we also considered the possible impact of Chinese immigrants, as well as the course of the infection vs. the density of the local population.

## DATA SOURCES

**Fig. 3:**
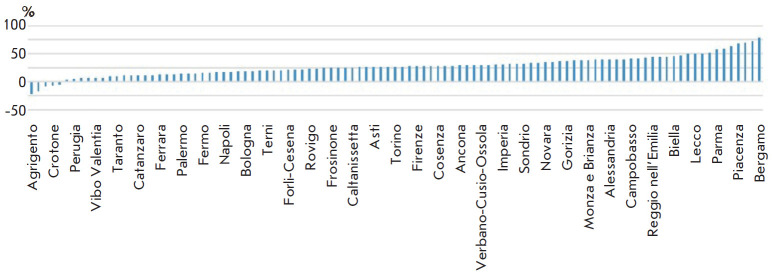
Percentage of variation of deaths across the provinces of Italy in the period under consideration (February 15 – April 15) – We present the extremes; the details are in *Table 1*

**Fig. 4:**
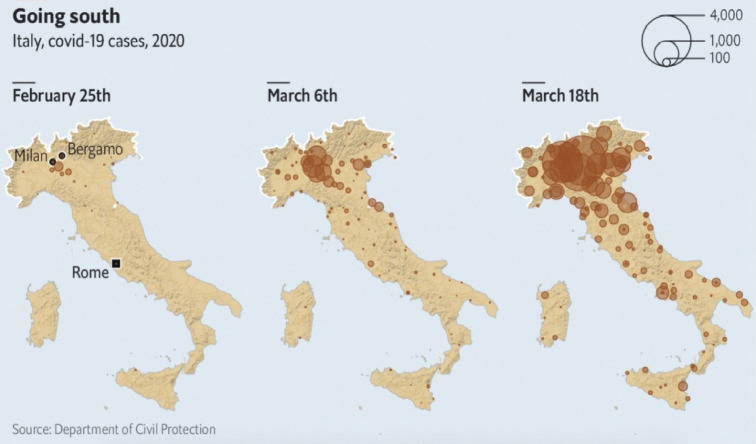
Covid-19 map spread in Italy. https://www.economist.com/europe/2020/03/19/italy-is-overtaking-chinaas- the-country-worst-hit-by-covid-19

**Fig. 5:**
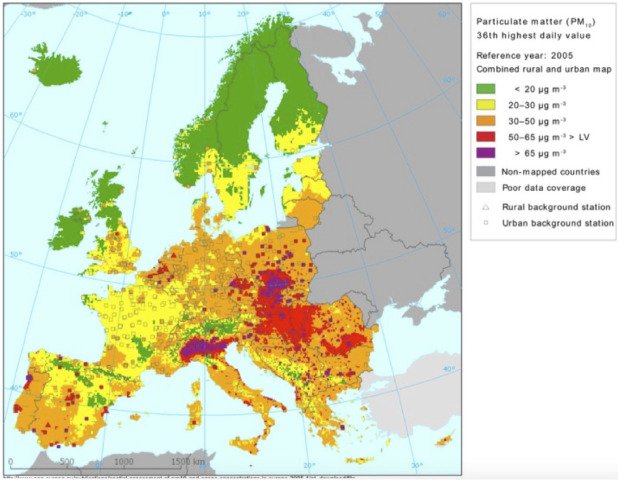
PM-10 contamination in Europe https://commons.wikimedia.org/wiki/File:PM10_in_Europe.png

We used data available in several public databases. Table 1 shows the distribution of the 2020 death rate compared with previous years as reported by ISTAT, the Italian Institute of statistics. The increase is particularly notable in the north, specifically in Lombardy [1]. Graphic is reported in Fig. 3. Data from Chinese immigration in Italy are also from ISTAT, which also provided data on the local population. Data on the infection incidence (https://github.com/pcm-dpc/COVID-19) come from the official GitHub repository of the Italian government [16] and is represented in Fig. 4. The data on infections and the death rate in India come from publicly available sources and reports of the death rate in the potentially affected regions of India as compared to the rest of the country (Table 2). Data regarding the PM-10 concentration in Italy were retrieved from the repository of ISPRA (Istituto Superiore per la Protezione e la Ricerca Ambientale/Italian Superior Institute for Protection and Environmental Research) [17]. Figure 5 shows the level of PM-10 concentration in Europe. It shows how its concentration was increased over the Padana landscape [18].

## STATISTICAL ANALYSIS

**Table 1:** Variation of the % of deaths in the period under consideration (Feb. 15 – Apr. 15) with respect to the same period in 2019

Province	Variation, %
Agrigento	-22.22
Cagliari	-16.67
Matera	-7.69
Crotone	-6.45
Catania	-5.56
Roma	3.94
Perugia	5.15
Arezzo	6.60
Lecce	6.72
Vibo Valentia	7.14
Ravenna	7.30
Foggia	9.16
Taranto	9.46
Messina	10.81
Sassari	10.93
Catanzaro	11.11
Teramo	11.11
Potenza	11.49
Ferrara	12.28
Salerno	12.98
Barletta-Andria-Trani	13.27
Palermo	14.04
Pisa	14.12
Siena	14.17
Fermo	15.00
Belluno	15.65
Venezia	17.41
Napoli	17.57
Brindisi	17.90
Trapani	18.95
Bologna	19.02
Macerata	19.32
Verona	19.47
Terni	20.00
Bari	20.15
Livorno	20.29
Forli-Cesena	21.33
Grosseto	21.54
Lucca	21.62
Rovigo	22.77
Oristano	23.58
Varese	24.33
Frosinone	24.49
Genova	24.65
Pistoia	24.79
Caltanissetta	25.00
Ascoli Piceno	25.53
Savona	25.61
Asti	26.46
La Spezia	26.55
Como	26.59
Torino	26.88
Pescara	26.88
Modena	27.51
Firenze	27.66
L’Aquila	28.00
Padova	28.03
Cosenza	28.05
Reggio di Calabria	28.26
Viterbo	28.38
Ancona	28.68
Massa Carrara	28.84
Vicenza	28.92
Verbano-Cusio-Ossola	28.99
Udine	29.41
Cuneo	30.02
Imperia	31.17
Nuoro	31.73
Treviso	31.88
Sondrio	32.34
Pordenone	33.33
Milano	33.80
Novara	34.73
Rimini	34.85
Chieti	36.36
Gorizia	36.84
Vercelli	37.61
Avellino	38.20
Monza e Brianza	38.86
Siracusa	39.34
Sud Sardegna	39.58
Alessandria	39.64
Latina	40.00
Isernia	40.00
Campobasso	40.82
Benevento	41.67
Trento	42.72
Reggio nell’Emilia	43.48
Mantova	43.77
Enna	44.78
Biella	45.48
Aosta	47.65
Pesaro e Urbino	49.56
Lecco	50.17
Pavia	50.51
Ragusa	51.85
Parma	56.97
Caserta	59.26
Brescia	64.25
Piacenza	68.57
Lodi	70.13
Cremona	71.93
Bergamo	78.77

Note: the province of Bolzano is not reported.

To analyze the data, we used the non-parametric Spearman’s Rank correlation coefficient [19]. Such a method includes no assumption on the underlying data, apart from being at least on an ordinal scale, which is always the case in our analyses. As the threshold of significance, we considered an α-level of 0.05, as customary. In the case where multiple hypotheses were being considered, we applied the Bonferroni correction [20]. In the case of an analysis of multiple factors, we used ANOVA [21]: again, considering the α-level mentioned above.

**Table 2:** Data about Covid-19 mortality in India. https://www.mohfw.gov.in/

S. No.	Name of State/UT	Total Confirmed cases	Cured/Discharged/Migrated	Deaths
1	Andaman and Nicobar Islands	33	33	0
2	Andhra Pradesh	2407	1456	50
3	Arunachal Pradesh	1	1	0
4	Assam	101	41	2
5	Bihar	1262	475	8
6	Chandigarh	191	51	3
7	Chhattisgarh	86	59	0
8	Dadar Nagar Haveli	1	0	0
9	Delhi	10054	4485	160
10	Goa	29	7	0
11	Gujarat	11379	4499	659
12	Haryana	910	562	14
13	Himachal Pradesh	80	44	3
14	Jammu and Kashmir	1183	575	13
15	Jharkhand	223	113	3
16	Karnataka	1147	509	37
17	Kerala	601	497	4
18	Ladakh	43	24	0
19	Madhya Pradesh	4977	2403	248
20	Maharashtra	33053	7688	1198
21	Manipur	7	2	0
22	Meghalaya	13	11	1
23	Mizoram	1	1	0
24	Odisha	828	220	4
25	Puducherry	13	9	1
26	Punjab	1964	1366	35
27	Rajasthan	5202	2992	131
28	Tamil Nadu	11224	4172	78
29	Telengana	1551	992	34
30	Tripura	167	85	0
31	Uttarakhand	92	52	1
32	Uttar Pradesh	4259	2441	104
33	West Bengal	2677	959	238
Total number of confirmed cases in India		96169	36824	3029

## RESULTS

**Chinese Immigration**

The presence of immigrants from China in Italy (https://www.tuttitalia.it/statistiche/cittadini-stranieri/ repubblica-popolare-cinese/) is not a factor in the spread of the virus [22]. In fact, in 2019, the number of Chinese present in Milan was 40,438 (1.25% of the total population), whilst in Rome their number was 22,815 (0,52%). The provinces with the highest percentage of increase in deaths had the following numbers: Bergamo 4,488 (0.40%), Cremona 1,362 (0.35%), and Lodi 757 (0.33%).

**Population Density**

Social proximity does not appear to affect the contagion and the death rate in Italy. We found no significant nonparametric correlation between the density of the population and the increase in mortality with respect to the last five years average (the p values are 0.083 and 0.071 respectively, indeed not significant), or between density and infection spread (0.17; again, absolutely not significant).

**Influence of the PM-10 level**

The PM-10 appears to have an effect considering the number of days above the threshold in Italy and, in particular, in Lombardy. There is a correlation of 0.40 with the number of deaths in 2019 (p < 10-4) and a correlation of 0.38 with the 5-year average of number of deaths (p < 10-3). There is also a correlation of 0.41 with the percentage of infected people (p < 10-4). However, if we consider together the effects of the distance from Seveso and the presence of PM-10 in a ranked ANOVA, we observe that the distance from Seveso retains its significance (t = -15.57, p < 10-8), while the presence of PM-10 does not.

**Distance from Seveso and Bhopal**

**Table 3:** Revealed statistically significant patterns of the significance of factors affecting the level of infection and mortality from COVID-19

Country	Possible cause	Possible effect, %	Spearman’s Rank Correlation
Italy	Distance from Seveso	Variation of death over 2019 Variation of death over 5-year average Infection	-0.82 (p < 10^-24^) -0.83 (p < 10^-25^) -0.88 (p < 10^-32^)
	Number of days of PM-10 over threshold	Variation of death over 2019 Variation of death over 5-year average Infection	0.40 (p < 10^-4^) 0.38 (p < 10^-3^) 0.41 (p < 10^-4^)
India	Distance from Bhopal	Deaths due to COVID-19 Infection	-0.52 (p < 10^-2^) -0.36 (p < 0.05)

The distance from Seveso appears to be a determining factor (Fig. 3). In terms of increase in deaths with respect to 2019 we found a very strong correlation: -0.82 (p < 10-24), whilst with respect to the average of the last five years it was -0.83, (p < 10-25). In terms of the percentage of infected population, the correlation is even higher, at -0.88 (p < 10-32). In summary, the closer to Seveso the analyzed sites were, the higher the rate of infected population and Covid- 19-related deaths were. In India, the correlation be tween distances from Bhopal is significant on both the reported percentage of deaths due to coronavirus (-0.52, p < 0.01) and of infected people (-0.36, p < 0.05) (Table 3).

## DISCUSSION

The possibility of a toxic gas escape that occurred 40 years ago playing a role in the increased incidence of complicated clinical courses in the recent Covid-19 infection is an intriguing, albeit difficult to demonstrate, hypothesis. As a result of both accidents, two different toxic gases were released, but both gases were characterized by high carcinogenicity [7, 11, 12, 13, 14, 15, 23, 24]. An increased mortality rate from COVID-19 was observed in all regions potentially exposed to the gases spread by the winds prevailing at the time of the accident.

An increased mortality rate due to the Covid-19 infection was witnessed in all the regions potentially touched by the gas leaks. This is also intimated in the observation of the possible effects of the winds active in those particular times of the year. This death rate increase was particularly striking in Lombardy, a fact that continues to require a plausible explanation. The particularly high virulence of the virus that affected the North of Italy was claimed as a possible reason for the high death toll [1]. Even if we assume that the better clinical course observed in the southern Italian regions was the result of heeding the lessons learned when the disease coursed through the northern parts of the country, the mortality rate difference remains hard to explain.

The possible detrimental effects of the PM-10 pollution has been invoked as a negative factor that has aided a more aggressive clinical course of the epidemic due to its chronic irritative impact on the respiratory system [25]. However, as we noted above, this hypothesis is somewhat contradicted by the observation that the impact of the epidemic in California has been definitely milder than it has been in New York, although the air concentration of PM-10 is much higher in California [26]. So, the detrimental effect of PM-10 pollution cannot be the sole reason for what was observed in Lombardy.

Other claims refer to the presence of immigrants from China. The available data from ISTAT show that on January 1, 2019, the number of Chinese present in Milan was higher than that in Rome. However, the provinces with the highest increase in deaths had lower numbers of Chinese immigrants. There are also claims that social proximity increases the contagion rate and, consequently, the death rate. However, we found that density did not push the mortality rate upward as relates to 2019 and to the last five years. Also, if we consider the number of infections, in this case there is also no significant correlation.

We hypothesize that the fallout of the Seveso accident – perhaps in addition to the detrimental effects of air pollution – would have acted synergically in Lombardy to make the clinical course of the coronaviral infection there particularly aggressive. It may have acted not only by predisposing residents, as a consequence of air pollution’s effect on the respiratory system of Lombard patients, to viral attacks, in particular to a significantly more aggressive course of the autoimmune reaction towards the alveoli the virus induces, but also through some gene-modifying mechanism that had taken place during the preceding 45 years and acted somehow by reinforcing the aforementioned autoimmune process. The other case, Bhopal, India, experienced an increased mortality rate as compared to the rest of the country. However, this difference, albeit significant, was not as striking as the one observed in Lombardy. We would venture that, in the region of Bhopal, the air concentration of PM-10 is not as significant as it is in the Padana landscape, which is a well-known site of significantly polluted air.

To support our claims, we used the robust Spearman’s Rank correlation coefficient. We considered first the relationship between the unequivocal number of variation of deaths in relationship to the previous years. The resulting value of the correlation of the distance from Seveso and the increase in deaths with respect to 2019 is impressive (-0.82, p < 10-24), and it is even more impressive with respect to the average for the last five years (-0.83, p < 10-25). We have also considered the relationship between distance to Seveso and the percentage of the infected population, and in this case the correlation is even higher -0.88 (p < 10-32).

For conclusiveness, we have also considered the claimed effect of PM-10, particularly by calculating the number of days above the safe threshold. We have noticed that, indeed, there is an impact, by far below the one related to the distance from Seveso (0.40, p < 10-4). The average number of deaths over 5 years (0.38, p < 10-3) and the percentage of those infected with COVID-19 (0.41, p < 10-4) also correlated with elevated levels of PM-10. We have built a ranked ANOVA to attempt to determine the joint contribution of the number of days of PM-10 above the threshold and the distance from Seveso. In performing such an analysis, we arrived at the conclusion that the distance from Seveso remains highly statistically significant, while the number of days of PM-10 above the threshold completely loses such significance.

To bolster our hypothesis, we turned our attention to the case of India, where we directly tested the presence of a correlation between the distance from Bhopal and the reported rate of infected and dead people from Coronavirus. The historical data on the total number of deaths and on the presence of PM-10 was not available to us. So, we had to rely only on the public data specific to the disease in 2020. In this case, we also found a statistically significant Spearman’s rank correlation between the distance from Bhopal and the percentage of infected people (-0.36, p < 0.05), as well as that of dead people (-0.52, p < 0.01).

## CONCLUSIONS

Our hypothesis, obviously, requires confirmation, perhaps through a study comparing certain genom ic characteristics of Lombardy longtime residents with those of relatively recent immigrants. As a matter of fact, a strikingly low presence of immigrants amongst the Covid-19 patients admitted to the ICUs of Lombardy hospitals has been observed [27], and a convincing explanation of that fact has yet to be provided. At the same time, we could not find in the scientific literature and statistical data direct evidence of increased mortality with seasonal influenza diseases in that region until the spring of 2020.

The technogenic catastrophe and the complicated course of COVID-19 in Lombardy may have something to do with the increased level of diabetes mellitus, oncological, and autoimmune disorders. Thus, population studies of mortality for 25 years since the accident in 1976, conducted by Consonni and colleagues, revealed increased additional mortality from diabetes among women in all areas of pollution, dependent on the degree of damage to the area [28]. According to available data, during the first 25 years after the technogenic accident (1976–2001), no increase in the total cancer mortality was detected throughout the affected areas. However, once the mortality rate was studied some 20 plus years after the explosion, an increase in cancer mortality was recorded in the area with the most severe pollution [28]. A similar correlation was observed with autoimmune diseases. In the affected areas, an inverse correlation was found between the level of immunoglobulin and dioxin in the blood plasma of adult patients [29]. At the same time, another study found an increase in the titers of antinuclear antibodies, an increase in the deposition of immune complexes, and a decrease in the number of natural killers in patients from the affected areas [30].

The half-life of dioxin in the body is 7–11 years. Since the disaster in Seveso occurred in 1976, the direct effect of dioxin can no longer be taken into account. Nevertheless, it is interesting to study the delayed effects of this substance on the human body. Since this manuscript considers the possible connection between residents of this particular area and the higher mortality rate from COVID-19, a next stage of this study could be the inclusion in the study sample of only the generation of people who directly experienced the accident of 1976 or moved to Seveso for 7–11 years until the half-life of dioxin expired. In a separate comparison group, it is possible to include the descendants of people who were affected by the accident and stayed in the territory. It is especially interesting to follow the individuals who survived the accident and their descendants who left for other regions of Italy and also suffered the new coronavirus infection. Unfortunately, at the moment (since the study is retrospective), such information is not available. Moreover, such data are not present either in open statistical data or in outpatient records. Therefore, a much larger resource is required for its systematization.

By focusing future research on the genomics and proteomics of affected patients in the area of technogenic disasters, especially young patients with a severe clinical course, it is possible not only to test the validity of our hypothesis, but also to predict the genetic determinants of individuals with a potentially worse prognosis of COVID-19. Such data could make the approach to treatment of COVID-19 more personalized, as well as identify risk groups that must be prioritized regarding vaccination, revaccination, and protection in terms of limiting social contacts.

## Abbreviations

COVID-19: COronaVIrus Disease 2019
PM-10: Particulate Matter-10 Microns or less
ISTAT: Italian National Institute of Statistics
ISPRA: Istituto Superiore per la Protezione e la Ricerca Ambientale/Italian Superior Institute for Protection and Environmental Research
